# Pharmacokinetic-guided magnesium prophylaxis in cardiac surgery: A randomized trial demonstrating guideline-level reductions in atrial fibrillation, accelerated recovery, and systemic cost savings

**DOI:** 10.2478/jccm-2026-0001

**Published:** 2026-01-30

**Authors:** Sarah Hamdy Elghareeb, Islam Taher, Ahmed Abd Al Ghany, Noha Mohamed Abdelaziz

**Affiliations:** Ain Shams University Faculty of Medicine, Cairo, Egypt

**Keywords:** atrial fibrillation prophylaxis, healthcare economics, ischemia-reperfusion injury

## Abstract

**Objective:**

To evaluate the efficacy, safety, and cost-effectiveness of a perioperative magnesium (Mg) sulfate protocol in reducing postoperative atrial fibrillation (AF) incidence and ICU resource strain following cardiac surgery.

**Methods:**

Design: Double-blind, single-center randomized controlled trial (RCT). Setting: Tertiary-care academic hospital. Participants: 130 adults undergoing elective cardiac surgery, randomized to Mg sulfate (n=65) or placebo (n=65). Interventions: The Mg group received a pharmacokinetic-guided regimen: 2 g intravenous bolus post-cardiopulmonary bypass, followed by 1 g/h infusion for 5 hours, then 200 mg/h for 19 hours, and oral supplementation (I g every 8 hours) for one week post-discharge. The placebo group received equivalent saline infusions and oral placebo.

**Results:**

Primary outcome: AF incidence was 18.5% in the Mg group vs. 41.5% in placebo (unadjusted RR=0.45, 95% CI: 0.25–0.81; p=0.007). Secondary outcomes: Mg shortened ICU stay by 1.4 days (p<0.001), reduced mechanical ventilation duration by 3.2 hours (p<0.001), and demonstrated comparable safety profiles for hypotension and renal impairment. Subgroup analysis: CABG patients showed 65% risk reduction (OR=0.35, p=0.01). Cost-effectiveness: ICU stay reduction projected $3,500 savings per patient.

**Conclusions:**

Perioperative Mg sulfate significantly reduces AF incidence, accelerates recovery, and lowers healthcare costs, supporting its integration into standardized postoperative protocols. This trial provides Level I evidence for Mg as a guideline-recommended intervention. These findings are promising and support the integration of Mg into standardized postoperative protocols; however, they require confirmation in larger, multicenter studies.

## Introduction

Atrial fibrillation (AF) remains the most common complication following cardiac surgery, affecting 25–40% of patients and contributing to prolonged hospitalization, stroke, and increased mortality [1,2,3]. Despite advances in surgical techniques and pharmacotherapy, optimal prophylaxis remains contentious [4]. Hypomagnesemia, frequently observed post-cardiopulmonary bypass, correlates strongly with arrhythmogenesis due to magnesium’s critical role in myocardial electrophysiology [5,6]. Magnesium stabilizes myocardial membranes, modulates calcium influx, and suppresses inflammation; all mechanisms implicated in AF pathogenesis [7,8]. While prior trials have explored Mg’s antiarrhythmic potential, heterogeneity in dosing regimens and patient populations has yielded conflicting results [9,10]. Notably, recent meta-analyses report null effects, potentially due to critical protocol variations e.g. subtherapeutic dosing or inclusion of off-pump surgeries [11,12]. This RCT evaluates a pharmacokinetic-guided perioperative Mg protocol. Unlike prior meta-analyses with heterogeneous dosing [11,12], this trial uses a targeted regimen (2g bolus post-CPB + infusion) to maintain therapeutic Mg levels (>2.5 mg/dL), based on pharmacokinetic studies [13].

## Methods

### Study Design and Population

This prospective, double-blind RCT (ClinicalTrials.gov ID: NCT06675500) enrolled 130 adults (18–70 years) undergoing elective cardiac surgery (CABG, valve replacement, or combined procedures) at Ain Shams University Hospitals between November 2024 and April 2025. Exclusion criteria included renal dysfunction (creatinine ≥1.8 mg/dL without adjustment for eGFR), any history of atrial fibrillation (permanent or paroxysmal), preoperative arrhythmias, emergency surgery, or redo operations. No patients had permanently implanted pacemakers or ICDs, as these were exclusion criteria. All surgeries were performed on-pump. Ethical approval was obtained from the Faculty of Medicine, Ain Shams University’s Institutional Research Ethics Committee (FMASU R255 /2024), and written informed consent was secured preoperatively. This trial adheres to CONSORT 2010 guidelines. The full checklist is available in Supplementary Figure 1.

### Intervention

Randomization used computer-generated variable blocks (sizes 4–6) stratified by surgery type (CABG vs. valve), with allocation concealment via sealed opaque envelopes opened post-anesthesia induction.

Blinding was maintained through pharmacy-prepared identical solutions (magnesium sulfate vs. 0.9% saline in matching bags) and tablets (magnesium oxide vs. inert placebo). Only the pharmacy team had access to randomization records. Outcome assessors were masked to group assignment throughout data collection.

No magnesium was administered during CPB or in cardioplegia solutions. All patients received cold blood cardioplegia (Buckberg solution; magnesium-free).

**Mg Group (n=65):** Received 2 g Mg sulfate intravenously immediately after weaning from cardiopulmonary bypass, followed by 1 g/hour for 5 hours, then 200 mg/hour for 19 hours. Oral Mg supplementation (1 g/8 hours) was continued for one week post-discharge. The Mg regimen (2 g bolus post-CPB, 1 g/hour infusion) was selected to maintain therapeutic levels (2.5–3.5 mg/dL) while minimizing hypotension risk, as validated in prior pharmacokinetic studies (13)

**Control Group (n=65):** Received equivalent volumes of 0.9% saline at matched timepoints and inert placebo tablets. No magnesium supplementation was permitted in controls during the entire study period, including CPB. Serum magnesium levels were monitored in both groups to confirm protocol adherence.

All patients resumed their preoperative beta-blocker regimen (bisoprolol 5 mg daily or propranolol 40 mg every 8 hours) within 24 hours postoperatively unless contraindicated (hemodynamic instability, heart rate <60 bpm, or SBP <100 mmHg). Adherence was confirmed in 98.5% of patients (64/65 per group), with one control patient requiring dose reduction due to transient hypotension. No amiodarone or other antiarrhythmics were administered perioperatively per exclusion criteria.

### Outcomes

**Primary Outcome**: Incidence of AF, AF was defined as an episode lasting > 30 seconds, confirmed via 12-lead ECG or continuous telemetry during hospitalization.

**Secondary Outcomes:** ICU/hospital length of stay (LOS), mechanical ventilation duration, postoperative renal impairment (creatinine ≥1.8 mg/dL), and complications (rebleeding, stroke, hypotension).

### Statistical Analysis

Based on published meta-analyses reporting AF incidence of 30–40% in controls following cardiac surgery (Cook et al., 2013 [11]; Klinger et al., 2015 [12]) and anticipating a 50% relative risk reduction (RRR) with our optimized Mg protocol (higher bolus dose, post-CPB initiation), we estimated a medium effect size (h=0.50). This RRR assumption was derived from:

Pharmacokinetic data confirming serum Mg levels >2.5 mg/dL reduce AF risk by 45–60% (Fairley et al., 2015 [6]; Shiga et al., 2004 [13])

Prior positive RCTs using similar high-bolus regimens (2–3g) showing 54–60% RRR (Miller et al., 2005 [14]; Kaplan et al., 2003 [15])

Using PASS 15, with power=80% and α=0.05, a sample size of 65 patients per group (total N=130) provides 85% power to detect an absolute risk reduction of 20% (control: 40%, Mg: 20%). This aligns with RCTs detecting similar effects in cardiac surgery cohorts (Gu et al., 2012; N=60–100/group [9]). Continuous variables were analyzed using independent t-tests or ANCOVA (adjusted for covariates); non-parametric data employed Mann-Whitney U tests. Categorical outcomes used chi-square or Fisher’s exact tests. Multivariable logistic regression adjusted for age, body mass index (BMI), chronic obstructive pulmonary disease (COPD), diabetes status, ejection fraction (EF), surgery type, and left atrial size (where available). Sensitivity analyses included propensity score matching (PSM) and Bayesian posterior probability estimation.

## Results

### Baseline Characteristics

Groups were well-matched in demographics, comorbidities, and surgical profiles. Mean age was 58.1±10.3 (Mg) vs. 59.0±9.7 years (control, p=0.61). Hypertension (76.9% vs. 81.5%, p=0.52) and diabetes (43.1% vs. 38.5%, p=0.58) were comparable. Preoperative EF (52.5% vs. 51.0%, p=0.29) and creatinine levels (1.1 vs. 1.0 mg/dL, p=0.41) showed no significant differences. CPB and cross-clamp times showed no significant intergroup differences (p>0.35), confirming balanced surgical complexity. Preoperative medication use was balanced between groups: beta-blockers (Mg 78.5% vs. placebo 80.0%, p=0.82), amiodarone (0% in both groups, as per exclusion criteria), and statins (Mg 75.4% vs. placebo 73.8%, p=0.84). No patients received amiodarone perioperatively. (Supplementary Table 1).

### Primary Outcome: Atrial Fibrillation Incidence

AF incidence was 18.5% (Mg) vs. 41.5% (placebo); unadjusted relative risk reduction=55% (95% CI: 32–71%, p=0.003 by chi-square). The Mg group exhibited a 55% relative risk reduction in AF incidence (18.5% [12/65] vs. 41.5% [27/65], adjusted OR=0.38, 95% CI: 0.18–0.79, p=0.007) (Table 1). This effect remained robust after adjusting for age, diabetes status, EF, and surgery type. Subgroup analysis demonstrated enhanced efficacy in CABG patients (OR=0.35, 95% CI: 0.15–0.82, p=0.01), potentially linked to Mg’s mitigation of ischemia-reperfusion injury (Table 1). The observed effect size exceeded assumptions (absolute reduction 23%, relative reduction 55%), confirming adequate power.

**Table 1.** Postoperative Outcomes

**Parameter**	**MG Group**	**Control Group**	**p-value**	**Statistical Test**
Atrial Fibrillation	12 (18.5%)	27 (41.5%)	0.007	Multivariable logistic regression (adjusted for age, EF)
ICU Stay (days)	2.1 ± 0.8	3.5 ± 1.2	<0.001	ANCOVA (adjusted for surgery type)
Hospital Stay (days)	6.2 ± 1.5	7.8 ± 2.1	<0.001	ANCOVA (adjusted for age, EF)
Ventilation Time (hrs)	8.4 ± 3.1	11.6 ± 4.3	<0.001	Quantile regression (median)
Renal Impairment	4 (6.2%)	9 (13.8%)	0.12	Generalized linear model (log-binomial)
Rebleeding	5 (7.7%)	9 (13.8%)	0.24	Fisher’s exact test
Open chest	3 (4.6%)	5 (7.7%)	0.47	Fisher’s exact test
Stroke	1 (1.5%)	2 (3.1%)	0.56	Fisher’s exact test
Hypotension (SBP <90 mmHg)	8 (12.3%)	10 (15.4%)	0.61	Chi-square

Magnesium Levels Over Time				
Timepoint	MG Group (mg/dL)	Control Group (mg/dL)	p-value	Statistical Test
Post-CPB	2.8 ± 0.4	1.9 ± 0.3	<0.001	Linear mixed-effects model
ICU Arrival	3.1 ± 0.5	2.0 ± 0.4	<0.001	Linear mixed-effects model
24 Hours Post-Op	3.4 ± 0.6	2.1 ± 0.5	<0.001	Linear mixed-effects model

Subgroup Analysis by Surgery Type				
Surgery Type	AF Incidence (MG)	AF Incidence (Control)	Adjusted OR (95% CI)	p-value
CABG	10/50 (20.0%)	22/53 (41.5%)	0.35 (0.15–0.82)	0.01
AVR	1/8 (12.5%)	3/6 (50.0%)		
MVR	1/5 (20.0%)	2/4 (50.0%)		
AVR/MVR Pooled	2/13 (15.4%)	5/10 (50.0%)	0.22 (0.04–1.25)	0.09
Combined	0/2 (0%)	0/2 (0%)		

Isolated AVR and MVR cases pooled due to sample size constraints; ‘Combined’ represents CABG + valve procedures. Subgroups: CABG (isolated), AVR (isolated aortic valve), MVR (isolated mitral valve). Multivariable logistic regression (adjusted for age, diabetes status, ejection fraction, and surgery type)

### Secondary Outcomes

**ICU and Hospital Stay:** Mg shortened ICU stay by 1.4 days (2.1±0.8 vs. 3.5±1.2 days, p<0.001) and hospital stay by 1.6 days (6.2±1.5 vs. 7.8±2.1 days, p<0.001), independent of surgery type or EF (Table 1).

**Ventilation Time:** Mechanical ventilation duration was 8.4±3.1 hours (Mg) vs. 11.6±4.3 hours (control) (mean difference -3.2h, p<0.001). Concurrent reductions in opioid and sedative requirements were observed (e.g., propofol doses: Mg 450±120 mg vs. control 620±180 mg, p=0.01), faster hemodynamic stabilization, and lower opioid consumption (fentanyl equivalents: Mg 750±250 μg vs. control 980±320 μg, p=0.003). (Table 1)

**Renal Impairment**: A nonsignificant trend favored Mg (6.2% vs. 13.8%, p=0.12), aligning with Mg’s vasodilatory and anti-ischemic renal protective effects. (Table 1) (16)

### Magnesium Levels

Serum Mg levels were significantly elevated in the Mg group at all timepoints: post-CPB (2.8±0.4 vs. 1.9±0.3 mg/dL, p<0.001), ICU arrival (3.1±0.5 vs. 2.0±0.4 mg/dL, p<0.001), and 24 hours postoperatively (3.4±0.6 vs. 2.1±0.5 mg/dL, p<0.001) (Table 1). Linear mixed-effects models confirmed sustained supratherapeutic Mg levels (p<0.001 for time-group interaction). Placebo group levels remained physiologically low (1.9–2.1 mg/dL), confirming no protocol deviations.

### Subgroup and Interaction Analyses

Mg’s protective effect was consistent across subgroups (Table 2):

**Age ≥60:** OR=0.35 (95% CI: 0.14–0.87, p=0.02).
**Diabetes:** OR=0.41 (95% CI: 0.19–0.91, p=0.03).

**Table 2.** Interaction Effects on AF Risk

**Subgroup**	**Adjusted OR (95% CI)**	**p-value**	**Interaction p-value**
Age ≥60 vs. <60	0.35 (0.14–0.87)	0.02	0.21
Diabetes vs. No DM	0.41 (0.19–0.91)	0.03	0.45
CABG vs. Valve	0.38 (0.15–0.94)	0.04	0.18

After adjusting for covariates including diabetes status, no significant interactions were observed for age (p=0.21), diabetes (p=0.45), or surgery type (p=0.18), underscoring Mg’s broad applicability.

## Discussion

This RCT demonstrates that perioperative Mg sulfate reduces AF incidence and accelerates recovery in cardiac surgery patients. The 55% relative risk reduction compares favorably with beta-blockers (30–40%) and amiodarone (45–50%) in similar cohorts, [17,18] suggesting Mg may be a cost-effective adjunct to existing therapies.

Mg’s antiarrhythmic efficacy likely stems from its multifaceted actions. Mg inhibits inward calcium currents and enhances sodium-potassium ATPase activity, stabilizing atrial repolarization and reducing ectopic triggers [19]. Cardiac surgery induces systemic inflammation, elevating cytokines (e.g., IL-6, TNF-α) that promote atrial remodeling. Mg’s inhibition of NF-κB signaling mitigates this proarrhythmic milieu [20]. Mg counteracts reactive oxygen species (ROS) generated during ischemia-reperfusion, preserving mitochondrial function and reducing atrial fibrosis [21].

The safety profile of the pharmacokinetic-guided regimen was favorable. Despite concerns regarding magnesium-induced side effects, we observed no significant intergroup differences in the incidence of hypotension (12.3% vs. 15.4%, p=0.61) or clinically significant bradycardia. The observed trend towards reduced renal impairment in the Mg group (6.2% vs. 13.8%, p=0.12) may reflect a balance between its potential diuretic and vasodilatory effects on renal perfusion, warranting further investigation.

The reduction of in-hospital POAF is a significant outcome, as POAF is associated with increased short-term morbidity, longer hospital stays, and higher costs. Furthermore, emerging evidence suggests that POAF is associated with a heightened long-term risk of stroke, heart failure, and mortality, independent of traditional risk factors [22,23,24]. Therefore, an effective and safe prophylactic intervention like magnesium could have implications beyond the immediate postoperative period. However, our study was not designed to assess these long-term outcomes, and future studies with extended follow-up are needed to determine if reducing POAF with magnesium translates into a reduction in these longer-term risks.

The 41.5% AF rate in controls exceeds some meta-analytical benchmarks (typically 30–35%), likely reflecting our tertiary center’s complex case mix and exclusion of off-pump surgeries. Similarly, prolonged ICU/hospital stays align with institutional protocols prioritizing hemodynamic stability over rapid extubation in high-risk cohorts (e.g., combined CABG/valve procedures). These factors support external validity for centers managing similar high-acuity populations.

The 41.5% AF rate in controls aligns with reported incidences in studies excluding amiodarone prophylaxis and off-pump surgeries [3]. Our cohort’s mean age (58.5 years) and EF (51.8%) rule out extreme risk profiles as an explanation.

The pronounced benefit in CABG patients (OR=0.35, p=0.01) aligns with Mg’s role in attenuating ischemia-reperfusion injury, a contributor to AF driver in coronary surgery [25].

The shorter ICU and hospital stay in the Mg group translate to tangible cost savings critical in resource constrained settings. For instance, reducing ICU stay by 1.4 days could save approximately $3,500 per patient when calculated using U.S. healthcare benchmarks. Cost savings ($3,500/patient) were estimated based on institutional ICU costs ($2,500/day) multiplied by the observed ICU stay reduction (1.4 days) [26]. However, these projections may not fully reflect institutional cost variations across different healthcare systems.

Similarly, the 3.2-hour decrease in ventilation time lowers ventilator-associated pneumonia risk, further curbing morbidity [27]. The observed reduction in median ventilatory time with magnesium supplementation was unexpected. While magnesium can theoretically cause muscle weakness, the dose used in this study was targeted at anti-arrhythmic levels and did not cause clinically significant neuromuscular blockade. Potential explanations warranting further investigation include a reduction in post-operative catecholamine surge by magnesium, leading to improved hemodynamic stability facilitating earlier extubation, or an indirect effect mediated by the significant reduction in post-operative atrial fibrillation, potentially reducing sedation needs or hemodynamic instability related to AF episodes. However, the absence of electromyographic data limits definitive conclusions about respiratory muscle impact and this finding should be interpreted with caution as it was a secondary outcome and requires validation. While extubation times (Mg: 8.4±3.1 hrs; control: 11.6±4.3 hrs) exceeded guideline targets (<6 hrs for routine cases), this reflects our institutional protocol prioritizing hemodynamic stability in complex procedures (e.g., combined CABG/valve), and institutional protocol prioritized stability over rapid extubation in high-risk cases.

Despite concerns about Mg-induced hypotension, rates did not differ between groups (12.3% vs. 15.4%, p=0.61). This contrasts with earlier studies reporting vasodilation at higher doses (e.g., 5 g boluses) [13], suggesting that controlled infusions (e.g., 2 g over 1 hour) optimize safety. The nonsignificant renal protection trend (6.2% vs. 13.8%, p=0.12) may reflect Mg’s diuretic properties, counterbalanced by its vasodilatory effects.

Our positive results versus null meta-analyses [11,12] likely stem from protocol differences: 1) higher bolus dosing (2g vs. ≤1.5g), 2) post-CPB initiation coinciding with hypomagnesemia nadir, 3) exclusion of off-pump surgeries, and 4) sustained supratherapeutic levels (mean 2.8–3.4 mg/dL vs. ≤2.2 mg/dL).

This trial’s strengths include rigorous randomization and comprehensive sensitivity analyses. Limitations include its single-center design, which may affect generalizability, and the short follow-up period, which precludes assessment of long-term AF recurrence, mortality, and other major adverse cardiac events. The fixed dosing regimen warrants exploration of optimal Mg dosing (e.g., weight-based adjustments). Furthermore, while the reduction in ICU stay demonstrated significant cost savings using U.S. benchmarks ($3,500/patient), these projections may not fully reflect institutional cost variations across different healthcare systems. Finally, data on certain potential confounders, such as left atrial size, were incomplete.

## Conclusion

Perioperative Mg sulfate prophylaxis significantly reduces AF incidence, accelerates recovery, and lowers healthcare costs, supporting its integration into standardized postoperative protocols. These findings advocate for Mg’s inclusion in international cardiac surgery guidelines and confirm the value of a pharmacokinetic-guided approach. Confirmation in larger, multicenter trials is warranted. This study provides robust evidence supporting the use of perioperative magnesium sulfate to reduce postoperative atrial fibrillation and accelerate recovery in cardiac surgery patients. The favorable safety profile and significant cost savings support its integration into standardized postoperative protocols. These findings advocate for the inclusion of a pharmacokinetic-guided magnesium regimen in international cardiac surgery guidelines, though confirmation in larger, multicenter trials is warranted.
